# The Influence of the Internal Forces of the Buckling Modes on the Load-Carrying Capacity of Composite Medium-Length Beams under Bending

**DOI:** 10.3390/ma13020455

**Published:** 2020-01-17

**Authors:** Monika Zaczynska, Zbigniew Kolakowski

**Affiliations:** Department of Strength of Materials, Faculty of Mechanical Engineering, Lodz University of Technology, 90-924 Lodz, Poland; zbigniew.kolakowski@p.lodz.pl

**Keywords:** composite LC-beams, beams of medium length, internal forces, interactive buckling, Semi Analytical Method (SAM), Finite Element Method (FEM)

## Abstract

The distribution of the internal forces corresponding to the individual buckling modes of lip-channel (LC) beams is investigated using the Semi Analytical Method (SAM) and the Finite Element Method (FEM). Channel section beams made of 8-layered GFRP (Glass Fiber Reinforced Polymer) laminate with three different layer arrangements were considered. The effect of the internal forces on the non-linear first-order coefficients corresponding to the interactive buckling was also studied. Moreover, distributions of the internal forces corresponded to the loading, leading to structure failure for which the load-carrying capacity was determined. The results indicated a high influence of the *N_x_* internal force component on the buckling loads and load-carrying capacity of the LC-beams.

## 1. Introduction

Thin-walled beam structures are widely used as primary structural components in engineering practice. I-section and C-section beams are often utilised as basic elements in many applications. In most thin-walled beams, the load-carrying capacity of these structures is limited not only by their strength but also by their stability. Thus, the problem of loss of stability is a subject of interest among researchers.

The phenomenon of unexpected loss of the load-carrying capacity of bent C-beams was observed during experimental research [1,2]. Kolakowski et al. [3] interpreted this behaviour as the effect of the secondary global distortional-lateral mode. Studies were performed on steel beams with channel and lip-channel cross-sections, and various beam lengths. To identify this phenomenon, the semi analytical method (SAM) within the second order of non-linear approximation was used. This method was found to be very useful for the interpretation of the interaction of different buckling modes in the full load range. The proposed method facilitates the understanding of the phenomena occurring during coupled buckling. In [4], the majority of conclusions from [3] were confirmed for steel LC-beams. The beam lengths for which the interaction of the secondary global buckling mode affects load-carrying capacity the most were also determined.

In the authors’ view, there is a lack of works devoted to the influence of the secondary global distortional-lateral buckling mode on the interactive buckling and load-carrying capacity of LC-beams. A few studies can be found in [5] in which the authors used FEM to verify the results of SAM calculations. High agreement of results was obtained. In [6,7], numerical and experimental studies were carried out on steel channel section beams to analyse the distortional-global interaction buckling. The strength and also the local buckling of channel section and Z-section beams subjected to bending loading were investigated numerically and experimentally in [8]. The post-buckling behaviour of cold-formed channels subjected to axial compressive loading or to pure bending was investigated in [9], while composite C-beams subjected to the bending moment were the object of investigation in, among others, Gliszczynski and Kubiak [10], Jakubczak et al. [1], and Kubiak et al. [11]. In view of the above, it was decided to present here only the latest and most relevant publications on this subject.

Since the late 1980s, the Generalized Beam Theory (GBT) has been developed extensively. The indisputable advantage of this method is the ability to determine the contributions of different deformation modes in the full range of structural loading. At the same time, the following numerical methods devoted to the non-linear analysis of stability were developed: FSM (Finite Strip Method), FEM (Finite Element Method), cFEM (constrained Finite Element Method).

Studies on the application of the Direct Strength Method (DSM) in the analysis of the distortional failure of steel beams are presented in [12]. In [13], the interactive buckling of lipped channel (LC) beams simply supported at both ends and subjected to uniform major-axis bending was analysed using the Generalised Beam Theory (GBT). Special attention was paid to the interaction of numerous simple buckling modes, in particular to the effect of the distortional-global buckling mode. The development of the interactive buckling theory of thin-walled structures is discussed in [14]. A semi-analytical procedure based on Koiter’s approach was used in [15] to analyse the post-buckling response of compressed thin-walled members. In [16], the method of modal decomposition of thin-walled structures taking into account coupled buckling modes is presented. The latest state-of-the-art cold-formed steel columns and beams influenced by the interactive buckling phenomenon, namely, local–distortional (L–D), local–distortional-global (L–D–G), and distortional–global (D–G), using a design based on the Direct Strength Method (DSM), are presented in [17]. Moreover, the differences for columns experiencing D–G interaction and beams affected by L–D interaction are indicated.

In [18,19], the analysis of the post-critical equilibrium paths of LC columns and beams was performed using the non-linear Generalized Beam Theory (GBT). Three types of L–D buckling modes were considered in the study. The stability of the composite beams subjected simultaneously to compressive and bending loading, including the effects of the transversal shear deformation of the plates and out-of-plane warping of the beam cross-section was analysed in [20]. The post-critical behaviour of the regular convex polygonal cross-section (RCPS) tubes subjected to uniform compression was investigated in [21,22]. The Generalized Beam Theory (GBT) was applied in order to analyse the effect of the local–distortional (LD) mode interactions of the columns with “short-to-intermediate” lengths. In turn, the purpose of article [23] was to present a DSM-based design method, which summarises the current knowledge and developments on the subject, and anticipates future applications of this approach.

The concept of interactive buckling (i.e., coupled buckling), which considers the general asymptotic theory of stability, is of fundamental importance for theoretical considerations. Koiter’s theory is the most popular among all versions of the general non-linear theory, due to its general character and continuous development [15,24,25]. The non-linear stability of thin-walled members in the first order approximation of Koiter’s theory formulated by Byskov and Hutchinson is solved with the modified analytical-numerical method (ANM) presented in [26]. The analytical-numerical method (ANM) should consider also the second-order approximation of Koiter’s theory in the investigations of the post-buckling behaviour of elastic structures. The second-order post-buckling coefficients were estimated with the semi-analytical method (SAM) [27].

In the present study, a plate model (2D) of a structure is considered to analyse all buckling modes. The exact transition matrix method is applied, as well as the numerical method of the transition matrix using Godunov’s orthogonalization. To obtain differential equilibrium equations, the principle of virtual works considering: Lagrange’s description, full Green’s strain tensor for thin-walled plates and the second Piola–Kirchhoff’s stress tensor, is used. The interaction between all walls of the structure, the shear lag phenomenon and the effect of cross-sectional distortions are taken into account and discussed. The most important advantage of this method is the possibility of describitng the thin-walled structures in the full range of behaviours from global to local stability [26,27].

In this paper, the effect of the interactive buckling of the distortional–global and local–distortional buckling modes on the post-critical equilibrium paths and load-carrying capacity was taken into account. The dominant character of the internal force components was indicated using SAM. In this analysis, the change of the internal force distribution in the post-critical regime is studied. The main purpose of the work is to indicate which components of internal forces and which buckling mode have a dominant influence on interactive buckling. According to the authors, this is a novel approach in the literature on the subject. The comparison was performed for the load-carrying capacity and failure (within the Hashin failure criterion [28] and the elastic limit load-carrying capacity of the structure) of composite LC beams. The studies were performed for three LC medium-length beams, which differed in laminate stacking sequence. The length of the beams was chosen based on the results of previous works [3,4], to obtain the strongest buckling interaction.

## 2. Formulation of the Problem

Thin-walled prismatic composite beams were taken into consideration. It was assumed that the beams were composed of plates, connected along longitudinal edges, subjected to uniform major-axis bending moment and simply supported at both ends [2,3,4,5,25,27].

To take into account all buckling modes (global, local and coupled buckling), a plate model (i.e., 2D) of thin-walled structures was used. The exact geometrical relationships (i.e., full Green’s strain tensor) were adopted for each plate to analyse both out-of-plane and in-plane bending of the *i-*th plate [26,27]:

(1) $$\begin{array}{l} \varepsilon_{xi}=u_{i,x}+\frac{1}{2}(w_{i,x}^{2}+v_{i,x}^{2}+u_{i,x}^{2})\\ \varepsilon_{yi}=v_{i,y}+\frac{1}{2}(w_{i,y}^{2}+u_{i,y}^{2}+v_{i,y}^{2})\\ 2\varepsilon_{xyi}=\gamma_{xyi}=u_{i,y}+v_{i,x}+w_{i,x}w_{i,y}+u_{i,x}u_{i,y}+v_{i,x}v_{i,y} \end{array}$$

and:

(2) $$\kappa_{xi}=-w_{i,xx}\;\;\;\kappa_{yi}=-w_{i,yy}\;\;\;\kappa_{xyi}=-2w_{i,xy}$$

where *u_i_*, *v_i_*, *w_i_*—the components of the displacement vector of the *i-*th plate in the *x_i_*, *y_i_*, *z_i_* axis direction, respectively, and the *x_i_-y_i_* plane overlaps with the mid-plane before it buckles.

To solve the non-linear problem of structure stability, Koiter’s theory [26,27] was implemented. The displacement fields *U* and the sectional force fields *N* were extended to the power series for the dimensionless amplitude of the *r-*th mode deflection *ζ_r_*:

(3) $$\begin{array}{l} U\equiv (u,v,w)=\lambda U_{0}+\zeta_{r}U_{r}+\zeta_{r}^{2}U_{rr}+\ldots \\ N\equiv (N_{x},N_{y},N_{xy})=\lambda N_{0}+\zeta_{r}N_{r}+\zeta_{r}^{2}N_{rr}+\ldots \end{array}$$

where: *λ*—load factor, *U*_0_, *N*_0_—the pre-buckling (i.e., unbending) fields, *U_r_*, *N_r_*—the first-order non-linear fields, *U_rr_*, and *N_rr_*—the second-order non-linear fields of the displacement and the sectional force, respectively. The range of indices is [1,*J*], where *J* is the number of interacting modes. It is assumed that the summation is over the repeated indices.

In thin-walled structures with initial geometric imperfections, *Ū* (only the linear initial geometric imperfections corresponding to the shape of the *r-*th buckling mode, i.e., *Ū* = ζ_r_* *U_r_* are taken into account), the total potential energy, can be described by the following equation:

(4) $$\Pi =-\frac{1}{2}M^{2}{\bar{a}}_{0}+\frac{1}{2}\sum_{r=1}^{J}{\bar{a}}_{r}\zeta_{r}^{2}\left(1-\frac{M}{M_{r}}\right)+\frac{1}{3}\sum_{p}^{J}\sum_{q}^{J}\sum_{r}^{J}{\bar{a}}_{pqr}\zeta_{p}\zeta_{q}\zeta_{r}+\frac{1}{4}\sum_{r}^{J}{\bar{b}}_{rrrr}\zeta_{r}^{3}-\sum_{r}^{J}\frac{M}{M_{r}}{\bar{a}}_{r}\zeta_{r}^{*}\zeta_{r}$$

Next, the equilibrium equations corresponding to Equation (4) have the form:

(5) $$\left(1-\frac{M}{M_{r}}\right)\zeta_{r}+a_{pqr}\zeta_{p}\zeta_{q}+b_{rrrr}\zeta_{r}^{3}=\frac{M}{M_{r}}\zeta_{r}^{*}\;r\;=\;1,\;\ldots ,\;J$$

where:

*M*—magnitude of the applied bending moment;
*M_r_*—buckling moment of the *r-*th buckling mode;
*ζ_r_*—the dimensionless amplitude of the *r-*th buckling mode;
ζ_r_*—the dimensionless amplitude of the initial deflection of the *r-*th buckling mode.

The buckling modes *U_i_* are mutually orthogonal, and can be described in the following form: *σ*_0_ *l*_11_(*U_I_*,*U_K_*) = 0, (*I*,*K*) = [1,*J*], *I ≠ K* where *J* is the number of all relevant buckling modes that are considered to be crucial in the structural response. The coefficients *ā*_0_, *ā_r_*, *ā_pqr_* and ${\overset{¯}{b}}_{rrrr}$ can be calculated from the equations described in the literature [26,27].

The following notations are introduced in Equation (5):

(6) $$a_{pqr}={\bar{a}}_{pqr}/{\bar{a}}_{r}\;\;\;b_{rrrr}={\bar{b}}_{rrrr}/{\bar{a}}_{r}$$

In the semi-analytical method (SAM), approximate values of the *b_rrrr_* coefficients (5) are determined based on the linear buckling problem. This approach makes it possible to estimate precisely the values of the *a_pqr_* coefficients (5), according to the applied non-linear Byskov and Hutchinson theory [26,27]. It should be mentioned that *a_pqr_* coefficients (5) are the key factors affecting interaction. For a more detailed analysis see Appendix A.

A relative angle of rotation of the girder ends was determined as a function of the *M/M*_min_, through differentiation of the expression of potential energy (4) for *M/M*_min_ [26,27]:

(7) $$\frac{\alpha }{\alpha_{\min}}=\frac{M}{M_{\min}}\left[1+\frac{M_{\min}}{M{\bar{a}}_{0}}\sum_{r=1}^{J}\frac{M_{\min}}{M_{r}}{\bar{a}}_{r}\zeta_{r}\left(0.5\zeta_{r}+\zeta_{r}^{*}\right)\right]$$

where: *α*_min_—the minimum buckling angle of rotation of the beam subjected to pure bending, corresponding to the minimum value of the buckling moment *M*_min_.

For the initial geometrical imperfect structure with amplitude ζ_r_*, at the point *M_s_*, where the load parameter *M* obtains its maximum magnitude (the so–called theoretical load-carrying capacity), the Jacobian of the non-linear system of Equations (5) is equal to zero. The solution is based on controlling the angle of rotation increase (7) for the structures subjected to bending. In this work, a three-mode approach (i.e., *J* = 3 in Equation (5)) was applied. This means that a model with three degrees of freedom is assumed. Instead of the finite strip method FSM, the exact transition matrix method is used in SAM.

It was decided to continue the analyses using FEM to validate the proposed SAM model and verify the obtained post-buckling equilibrium paths and load-carrying capacities. This comparison allows the determination of the cases for which global secondary buckling should be considered. It is worth noticing that the cost (i.e., time) of the SAM calculation is more than 30 times lower than that of FEM. In addition, the semi-analytical method SAM allows for a much simpler analysis of the observed phenomena and their interpretation compared to FEM.

**Figure 1:**
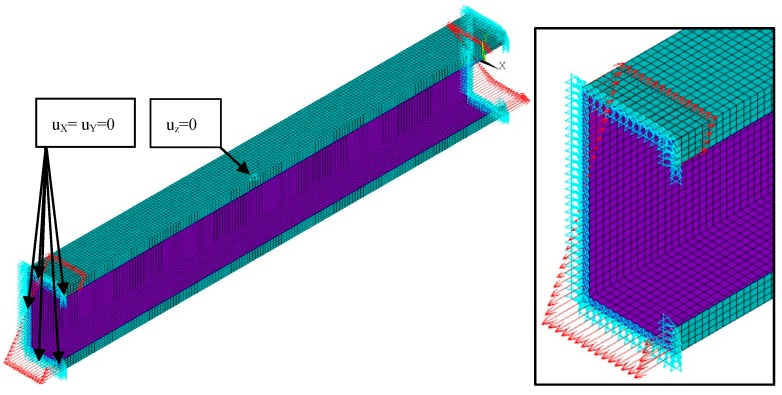
Discretized numerical model with applied type I boundary conditions.

**Figure 2:**
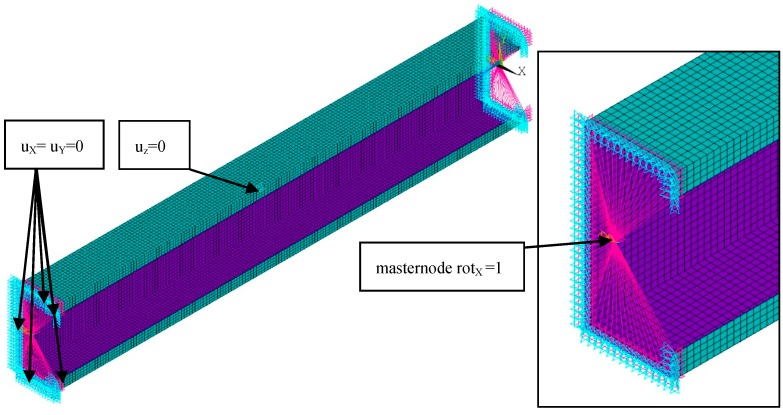
Discretized numerical model with applied type II boundary conditions.

The calculations were also performed using the finite element method (FEM) with the 18.2 ANSYS^®^ software version [29]. The composite beam was modelled using 10 754 SHELL181 elements and 65 434 degrees of freedom. SHELL181 is a 4-node element with six degrees of freedom at each node [29]. The size of the element was determined based on the convergence analyses and set to 3 mm. The boundary conditions were applied to ensure pure bending and simple support. To fulfil the condition of simple support, the displacements in the transverse directions at the ends of the beam (u_x_ = 0, u_y_ = 0 in Figure 1 and Figure 2) were removed. Moreover, the displacement in the longitudinal direction was set to zero at the mid-length point of the beam and the mid-width point of the web. The beam loading providing the pure bending was applied in two ways, as follows [5]:

- BC I—the beam was loaded by the bending moment generated from normal forces located at the nodes in both beam ends with different force magnitude (Figure 1). The force distribution corresponds to the stress distribution in the case of pure bending.
- BC II—the bending moment was applied by the displacement of the beam ends. The angle of rotation was applied in the “maternode” located at the centre of gravity of the cross-section and transferred to all nodes lying at both ends of the beam cross-sections (Figure 2). This method of load application corresponds to that used in SAM.

The bending moment and angle of rotation were determined separately for each type of load application (BC I and BC II). For BC I, the bending moment *M* was determined as the sum of forces acting on the beam and the distance from the neutral axis, while the angle of rotation α was determined based on the displacement of the flange in the beam’s support, according to the following equation:

(8) $$\alpha =arctg\frac{U_{zn}}{y_{max}}$$

where:

*U_zn_*—displacement in the *z*-direction of the point located on the flange, on the *y*-axis;
*y_max_*—maximum distance from neutral axis to the outer layer;

For BC II, the bending moment *M* was determined as the reaction (moment) of the applied load in the “masternode”, while the angle of rotation *α* was determined directly from the applied load. The solution differs for the two FEM models: for FEM I BC it is based on controlling the bending moment increase, while for FEM II BC on controlling the angle of rotation increase.

The studies were carried out for thin-walled beams of medium length equal to L = 500 mm with 1 mm wall thickness. Eight-layer symmetrical GFRP laminates with a thickness of individual ply equal to t_1_ = 0.125 mm (i.e., t = 8t_1_) were considered. 

**Figure 3:**
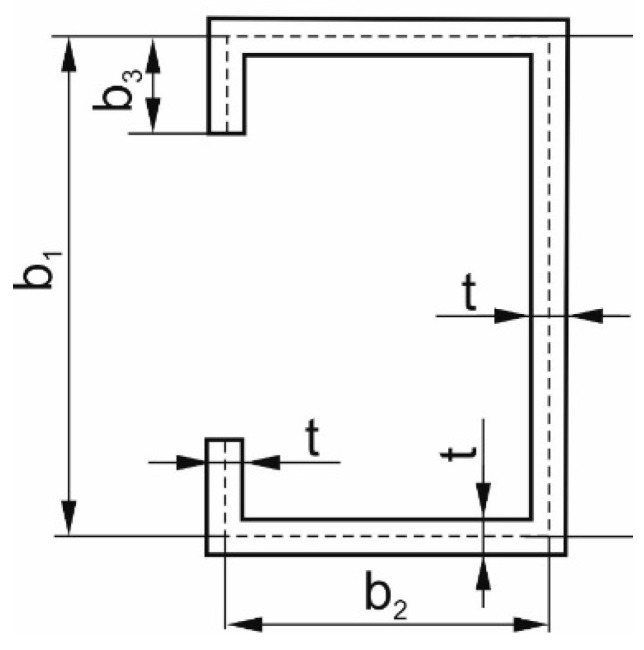
Cross-section of the LC beam.

The cross-section of the lip channel beam is presented in Figure 3 with dimensions listed in Table 1. Mechanical properties, such as elastic properties and strength limits, were determined in the main orthotropic directions, as listed in Table 2. Depending on fibre orientation, three different stacking sequences (i.e., three instances) were analysed (Table 3).

## 3. Results and Discussion

### 3.1. Linear Buckling Analysis

In the first stage, eigenbuckling analysis was performed for LC beams to determine the buckling stresses of the considered buckling modes.

The following indexes were introduced:

1—the primary global distortional–lateral buckling mode for *m* = 1 (*m* corresponds to the number of half-waves in the longitudinal direction);

2—the secondary global distortional–lateral buckling mode for *m* = 1;

3—local distortional buckling mode for *m* = 2;

4—local buckling mode for *m* > 2;

The interactive buckling of three modes was examined in this work.

Table 4 presents the critical stress values σ_i_ (i.e., eigenvalues) for the three layer arrangements considered, obtained from FEM and SAM. The lowest values of the bifurcation loads σ_i_ were obtained for: LC–1 for *m* = 2, LC–2 for *m* = 2, and LC–3 for *m* > 2 (for SAM) and for *m* = 1 (for FEM). Comparing the three analysed instances (LC–1, LC–2 and LC–3), the lowest values of the bifurcation loads for local and global modes were obtained for LC–1, while for LC–2 and LC–3 they are similar and larger by about 30% than for LC–1. For LC–1, the lowest values of the critical load σ_i_ were obtained for local and global modes, with σ_1_/σ_3_ = 1.1. Respectively, for LC–2 σ_3_/σ_1_ and LC–3 σ_4_/σ_1_ (SAM), σ_1_/σ_4_ (FEM) were below 1.05. Therefore, the magnitudes of critical load for each instance are close to one another. Based on these results, it can be concluded that the layer arrangement of the LC–1 beam is the least favourable for the considered instances due to the value of the critical loads.

In Table 5, the values of the critical moment *M*_min_ corresponding to the lowest critical load for each of the three instances, and also those corresponding to the *M*_min_ angle of rotation at the support *α*_min_ are listed. Comparing the three applied methods (FEM BC I, FEM BC II and SAM), a very high result accuracy was obtained for the two FE models. Some deviations can be noticed when SAM outcomes are taken into account. The magnitude of buckling stresses, bending moments and angle of rotation determined from SAM are up to 10% greater than from FEM. This difference between FEM and SAM results is due to the differences in the models in both methods. The number of degrees of freedom, three for SAM and over 65,000 for FEM, reveals that the results obtained from SAM are higher than FEM. The buckling modes (the so-called eigenvectors) corresponding to the analysed instances obtained from FEM are presented in Table 6. For each instance and buckling mode, the cross-sections of the buckling modes for the maximum deflection amplitude are also listed in Table 6. It should be mentioned that eigenvectors are determined in the increments of a unit. Comparing the individual buckling modes for each of the three considered instances LC–1, LC–2 and LC–3, it can be said that the buckling modes are practically identical for the given i = 1, 2, 3 in increments to a unit (i.e., deflections outside or inside for i = 3). For i = 4 (i.e., for the local mode for *m* > 2), the obtained buckling modes differ in the number of half-waves in the longitudinal direction. Therefore, for LC–1, there are ten half-waves, and 12 and 11 for LC–2 and LC–3, respectively.

As part of the analysis of the eigenvalue problems corresponding to the bifurcation loads, the *N_x_, N_y_* and *N_xy_* components of internal forces were determined. For a given critical load, the force components were determined in increments of a unit. Therefore, magnitudes of the internal forces for different bifurcation loads cannot be compared. Only force components for a given eigenvalue can be compared. 

**Figure 4:**
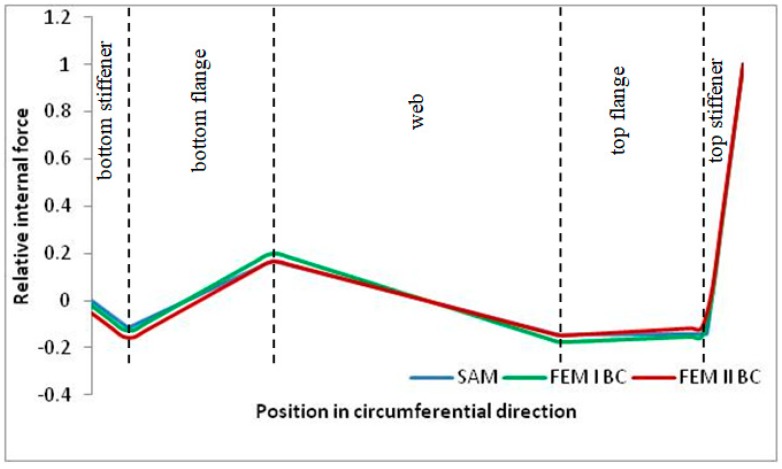
The distribution of *N_x_* internal force in LC–1 beam cross-section for the primary global distortional–lateral buckling mode.

Internal forces were determined numerically for the two analysed boundary conditions (FEM BC I, FEM BC II) and compared with the SAM results. The internal forces *N_x_* and *N_y_* were determined in half of the half-wave length, while *N_xy_* at the end of the beam. The distribution of internal forces *N_x_, N_y_* and *N_xy_* in the beam cross-section is presented in the charts dimensionless, by introducing the parameter “Relative internal force”. This parameter is defined as the ratio of a given internal force (*N_x_*, *N_y_* or *N_xy_*) to the maximum value of the internal force in the given instance and buckling mode. Taking into account the fact that in each analysed instance, the highest value is always reached by an internal force *N_x_*, the “Relative internal force” is defined as *N_x_*/*N_xmax_*, *N_y_*/*N_xmax_* or *N_xy_*/*N_xmax_*. This procedure allows the observation of the share of each internal force. Figure 4 presents indicative results with the distribution of the *N_x_* internal force for the primary global distortional–lateral buckling mode in the LC–1 beam cross-section. The force components are presented in the graphs as a force in the function of the cross-sectional length, as shown in Figure 4. The parameter is equal to zero at the beginning of the tensioned edge of the stiffener (bottom stiffener in Figure 1 for example) and gains the maximum value at the end of the compressed edge of the stiffener (top stiffener). For all force components and all modes for the three considered instances, good compatibility between the methods (i.e., FEM and SAM) can be observed. 

The evolution of the *N_x_* force component for i = 1 and instances LC–1, LC–2 and LC–3 are relatively small (below 0.2) for the tensioned bottom stiffener and flange. For the web, tensioned in the bottom part and compressed in the upper part, the internal force *N_x_* sign changes. At the same time, for the compressed top flange, it is almost constant (−0.2) and increases rapidly when the compressed edge reinforcement reaches the maximum value (value 1.0). The evolution of the internal forces *N_x_* for i = 3 and i = 4 are small for tensioned component plates and increase rapidly for the compressed flange and stiffener, for which they reach their maximum value. The evolution of the internal force *N_x_* for i = 2 is the most rapid. The results of the computations are presented in Table 7, Table 8 and Table 9. For each considered buckling mode and stacking sequence, the highest share of *N_x_* internal force is observed. Internal forces *N_xy_* constitute a maximum of 40% of the *N_xmax_* force, while the contribution of the *N_y_* internal force reaches a maximum value of 25% of the *N_xmax_* internal force. It should be emphasised that the highest shares of *N_y_* internal forces occur in the case of the lowest local buckling mode (i = 4). For other considered buckling modes, their contribution is negligible (up to 1.5% of internal force for the secondary global distortional–lateral buckling mode in the LC–2 beam). Similarities can be observed in the distribution of internal forces among the considered samples. 

### 3.2. Non-Linear Analysis

Next, a non-linear stability analysis was performed to determine the influence of the *N_x_, N_y_* and *N_xy_* internal force components on the critical equilibrium paths and on load-carrying capacity. In SAM, the buckling interaction occurs only within the first non-linear order of approximation. First-order non-linear coefficients (4) and the corresponding (through dependence (6a)) coefficients (5) are determined based on their eigenvalue modes (i.e., eigenvectors). For a more detailed analysis, see Appendix A. Determining the three index coefficients makes it possible to analyse the influence of each of these modes on the values of the coefficients, which is not possible as part of the eigenvalue problem.

To determine the effect of the most significant buckling modes, the values of *a_pqr_* coefficients were determined for each of the equations of equilibrium (5). In this analysis, only the three-mode approach was considered taking into account the interaction of the following buckling modes:

- Case 1—the interaction of buckling modes 1, 2 and 3;
- Case 2—the interaction of buckling modes 1, 2 and 4;

For three instances LC–1, LC–2 and LC–3.

For each instance, the largest three index coefficient *max(a_pqr_)* was selected from the three equations of equilibrium (5). Table 10 lists the index designations of these *a_pqr_* coefficients and (in parentheses) the elements dependent on dimensionless deflection parameters. Table 10 presents the coefficients which play a major role in the interaction, i.e., coefficients meeting the relationships 0.8*max(a_pqr_)* < *a_pqr_* < *max(a_pqr_)* and second-order coefficients satisfying the relationship 0.2*max(a_pqr_)* < *a_pqr_* < 0.8*max(a_pqr_)*. For *a_pqr_* coefficients, the following rule stands: e.g., coefficient *a_211_*, according to Equation (4), is equal to *a_211_ = a_112_ + a_121_ + a_211_*.

Comparing the results presented in Table 10, it can be observed that for LC–1, the coefficients at ζ_1_ζ _1_^2^ and ζ_2_ ζ _3_^2^ in energy always play the primary role (7), while coefficients ζ_1_ ζ _3_^2^ are secondary. For LC–3, there is no secondary member. For LC–2, the primary members are ζ_1_ζ _4_^2^ and ζ_2_ ζ _4_^2^, and ζ_2_ ζ _1_^2^ are auxiliary. Based on this comparison, it can be seen that the effect of the secondary global buckling mode (i = 2) is greater in case 1 than in case 2. These conclusions are valid for LC–1, LC–2 and LC–3.

In the non-linear analysis of *a_pqr_* coefficients, it was observed that they depend on the sum of three elements of integration, which rely among others on the internal force components *N_x_, N_y_, N_xy_* (for a more detailed analysis see Appendix A). Thus, the influence of these internal force components *N_x_, N_y_, N_xy_* on *a_pqr_* coefficients was analysed. For this purpose, the numerical reset for the individual forces’ component was calculated in a computer program. The following dimensionless ratios were introduced: I1=*a_pqr_* (IX = 0)/*a_pqr_*, I2 = *a_pqr_* (IY = 0)/*a_pqr_*, I3 = *a_pqr_* (IXY = 0)/*a_pqr_*, where the coefficients occurring in the counter are determined by adopting IX = 0, IY = 0 and IXY = 0 expressed by Equations (A4)–(A7), respectively.

The maximum dimensionless ratios I1, I2, I3 for three instances and two cases are shown in Table 11. It is evident that the *N_x_* component has the most significant influence on the values of coefficients *a_pqr_*, because its omission reduces the coefficients by at least 94%. If the *N_y_* component is omitted, this influence is at most 4%, and for *N_xy_* it is at most 7% of the value. This demonstrates the dominant influence of the *N_x_* component on the magnitude of *a_pqr_*.

Next, the post-critical analysis of equilibrium paths and load-carrying capacity was carried out. In the case of SAM, all coefficients *a_pqr_* of the system of Equation (5) were taken into account. The following magnitudes of initial imperfections were assumed: ζ_1_* = |1.0|, ζ_2_* = |1.0|, ζ_3_* = |0.1| and ζ_4_* = |0.1|. Similar to FEM, the amplitudes of the structure imperfections were set to 1 (for the global buckling modes, i.e., i = 1,2—w_oi_/t = 1) and 0.1 (for the local ones, i.e., i = 3,4—w_oi_/t = 0.1) for the thickness of the beam walls. The signs of the imperfections were chosen to obtain the lowest magnitude of load-carrying capacity [1,3,4,26].

In Table 12, the dimensionless load-carrying capacity related to the lowest critical load *M_s_/M*_min_ for two cases (i.e., case 1 and case 2) with the three-mode approach and three calculation methods is presented. In SAM, the interaction of the buckling modes for case 1 always provides lower load-carrying capacity *M_s_/M*_min_ than for case 2. This indicates a stronger distortional–local influence of mode i = 3 (for *m* = 2) on interactive buckling than the “pure” local mode i = 4 (for *m* > 2). In SAM, it is possible to separate all buckling modes as opposed to FEM, where it is virtually impossible. For three instances, lower magnitudes of the *M_s_/M*_min_ were obtained for FEM BC I and FEM BC II. The value of *M_s_/M*_min_ load-carrying capacity is always lower than the minimum critical moment. For SAM, the load-carrying capacity drops to about 20% for LC–1, LC–2 and LC–3, while for FEM and LC–1 it drops to 2%, for LC–2 to 9%, and for LC–3 to 11%.

Table 13 presents the post-buckling equilibrium paths for three instances where the lowest load carrying-capacity was obtained by the SAM method. According to Equation (7) on post-critical equilibrium paths, the lowest value of the bifurcation moment *M*_min_ and the corresponding angle of rotation at the support *α*_min_ should be taken as a reference value. According to Table 4, for LC–1 the lowest value of bifurcation moment corresponds to i = 3, for LC–2 it is also i = 3, and for LC–3, and it is i = 4 (for SAM). Table 13 presents a comparison of the *M/M*_min_ post-critical equilibrium paths as a function of the rotation angle on the support *α/α*_min_ for the three used methods. Moreover, in the equilibrium paths obtained from FEM, the load-carrying capacity was marked with dots, while the load leading to composite failure determined with the Hashin failure criteria was marked with “*x*”.

The post-critical equilibrium paths for FEM BC I and FEM BC II are close to each other in the considered ranges of *α/α*_min_ variability. The curves for SAM are well matched to the curves for FEM. Nevertheless, they reach their maximum value, i.e., the load-carrying capacity *M_s_/M*_min_, below the load-carrying capacity obtained from FEM, and then fall much more rapidly than in the case of FEM. It should be mentioned, however, that SAM is a lower-bound estimation of the structure’s load-carrying capacity. The average difference reaches a value close to 10% for LC–1 and LC–2, and the value of 5% for the LC–1 beam.

Using the Hashin failure criteria for the material properties given in Table 2, the maximum bending moments corresponding to the destruction of the first composite layer *M_H_* were determined using FEM for the considered instances. In Table 14, as in Table 12 for LC–1, LC–2 and LC–3, the dimensionless maximum loads referred to the lowest critical load value *M_H_/M*_min_ for two cases (i.e., case 1 and case 2) for three modal approaches. The post-critical equilibrium paths are very flat near the load-carrying capacity. This results in the *M_H_/M*_min_ values for the corresponding cases being similar to *M_s_/M*_min_ (Table 12 and Table 13). For LC–2, layer failure occurs immediately after reaching load-carrying capacity, while for the other two instances composite failure occurs before reaching the ultimate load. For each of the considered beams, failure occurred as a result of the matrix cracking under compression. 

Table 14, Table 15, Table 16 and Table 17 show the distribution of internal forces N_x_, N_y_ and N_xy_ corresponding to the maximum moments *M_H_/M*_min_ (Table 14) and the load-carrying capacity *M_s_/M*_min_ (Table 12) obtained for FEM BC I and FEM BC II. Solid lines denote the distributions for the maximum moment *M_H_/M*_min_, and dashed lines those for *M_s_/M*_min_. For each considered instance, the largest share of *N_x_* internal force is observed. Internal force *N_xy_* constitutes a maximum of 20% of the *N_xmax_* force, while the contribution of the *N_y_* internal force reaches a maximum value of 3% of the *N_xmax_* internal force. In this case, the *N_x_, N_y_* and *N_xy_* internal force distributions are, as expected, more non-linear than in the case of distributions corresponding to a linear eigenvalue problem. The *N_x_* and *N_xy_* internal force distributions corresponding to the first failure (detected with the Hashin failure criteria) and the load-carrying capacity are similar. Larger differences can be observed for the *N_y_* internal force distributions. The graphs of the *N_x_* internal force distribution, considering the three-mode approach for modes i = 1, 2, 3 and i = 1, 2, 4 are very similar, as expected for FEM. LC–2 may raise some doubts. It should be noted; however, that when the absolute values of forces are taken into consideration, these graphs are coincident, which corresponds to the critical symmetrical equilibrium path.

SAM, as used in this study, explained the phenomenon of rapid loss of load-carrying capacity for medium-length LC-composite beams. FEM enables the verification of the observed phenomena. This phenomenon is always associated with the influence of the secondary global distortional-lateral buckling mode with the primary global distortional–lateral and local modes. This interaction was analysed taking into account the components of internal forces *N_x_*, *N_y_* and *N_xy_*. FEM was used to verify the observed phenomenon and analyse it more precisely. In addition, FEM made it possible to compare the determined load-carrying capacity with the maximum moment determined based on the Hashin failure criteria and to illustrate the distribution of the internal forces for these load values. The linear and non-linear analysis shows a very large influence of the internal force component *N_x_* on the bifurcation loads and load-carrying capacity of the LC-beams.

## 4. Summary

This work analyses the distribution of internal forces corresponding to the individual buckling modes as part of the eigenvalue problem. It considers their effect on the non-linear first-order coefficients corresponding to the interactive buckling for the three-mode approach using the semi-analytical method (SAM) and the load-carrying capacity of the bent composite LC-beams. The results of the SAM analysis were verified by FEM for two types of boundary conditions of the LC-beams. Also, in the case of FEM, the distributions of the internal forces corresponding to the load-carrying capacity and load leading to the first failure determined based on the Hashin failure criteria were presented. The results of the analysis were presented for three-layer arrangements of composite beams of medium length.

Based on the conducted research, it can be concluded that:

- for LC–1 and LC–2 beams, the lowest buckling mode has a local–distortional character, while LC–3 has a global distortional–lateral buckling mode
- similarities occur in the distribution of the internal forces (*N_x_*, *N_y_* and *N_xy_*) for the analysed buckling modes among all samples
- the greater influence of the secondary global buckling mode (i = 2) occurs when the interaction of the primary and secondary global distortional–lateral buckling mode and distortional buckling mode (case 1) is considered
- the *N_x_* internal force has a primary effect on the magnitude of coefficient *a_pqr_*
- linear and non-linear analyses for all instances (LC–1, LC–2, LC–3) reveal the highest share of *N_x_* internal force
- the load-carrying capacity for all considered samples is lower than the critical bending moment
- the load-carrying capacity estimated by SAM is lower than that obtained from FE models
- the failure of the composite layer occurs before reaching load-carrying capacity (for LC–1 and LC–3) or just after reaching the ultimate loading (for LC–2)
- the distribution of the internal forces in the post-critical regime is more non-linear compared to distributions gained from linear analysis
- the discrepancy between SAM and FEM results is caused by the different number of degrees of freedom adopted in both methods

## Figures and Tables

**Table 1:** Dimensions of the beam.

b_1_ [mm]	b_2_ [mm]	b_3_ [mm]	t [mm]	L [mm]
80	40	10	1	500

**Table 2:** Mechanical properties of the GFRP composite.

E_1_ [GPa]	E_2_ [GPa]	G_12_ [MPa]	*v*_12_ [-]	T_1_ [MPa]	T_2_ [MPa]	S_12_ [MPa]	C_1_ [MPa]	C_2_ [MPa]
40	10	4	0.3	1250	43	112	620	140

**Table 3:** Layer arrangements.

Instances	Layer Orientation
LC–1	[45/−45/45/−45]s
LC–2	[45/−45/90/0]s
LC–3	[0/90/0/90]s

**Table 4:** Comparison of buckling stresses for considered buckling modes.

Methods	LC–1				LC–2				LC–3			
	σ_1_ *m* = 1	σ_2_ *m* = 1 s	σ_3_ *m* = 2	σ_4_ *m* > 2	σ_1_ *m* = 1	σ_2_ *m* = 1 s	σ_3_ *m* = 2	σ_4_ *m* > 2	σ_1_ *m* = 1	σ_2_ *m* = 1 s	σ_3_ *m* = 2	σ_4_ *m* > 2
	MPa	MPa	MPa	MPa	MPa	MPa	MPa	MPa	MPa	MPa	MPa	MPa
FEM BC I	39.1	142.8	30.8	56.9	42.8	213.6	41.1	56.0	40.7	246.2	43.7	42.0
FEM BC II	36.2	147.6	31.7	58.7	44.4	220.5	42.4	57.8	42.2	254.1	45.1	43.4
SAM	37.6	148.3	33.9	57.9 (*m* = 12)	46.9	219.8	45.7	57.3 (*m* = 13)	45.4	251.8	48.8	43.4 (*m* = 11)

**Table 5:** Comparison of buckling moment and angle of rotation.

Methods	LC–1		LC–2		LC–3	
	*M*_min_ [Nm]	*α*_min_ [-]	*M*_min_ [Nm]	*α*_min_ [-]	*M*_min_ [Nm]	*α*_min_ [-]
FEM BC I	155.93	0.01548	206.90	0.01293	205.30	0.01014
FEM BC II	155.93	0.01586	206.91	0.01337	206.00	0.01053
SAM	165.55	0.01704	223.17	0.01438	211.94	0.01078

**Table 6:** Buckling modes of the considered FRP beams.

Instance	Buckling Mode			
	i = 1	i = 2	i = 3	i = 4
LC–1	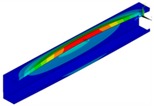	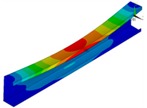	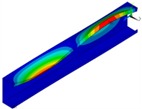	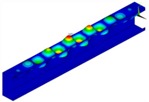
	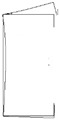	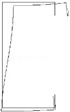	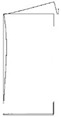	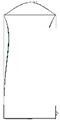
LC–2	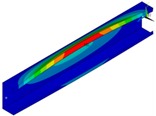	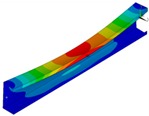	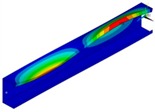	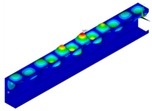
	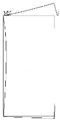	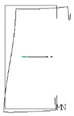	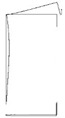	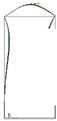
LC–3	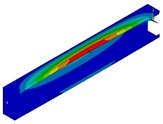	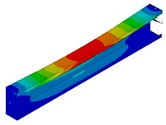	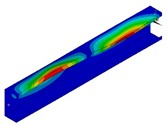	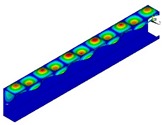
	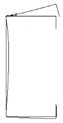	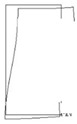	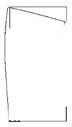	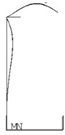

**Table 7:** The distribution of internal forces *N_x_, N_y_* and *N_xy_* for the LC–1 beam.

Index	*N_x_*	*N_xy_*	*N_y_*
i = 1	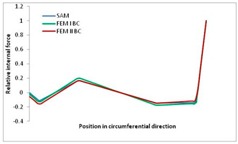	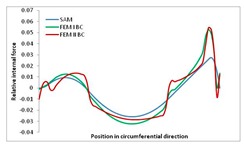	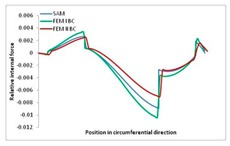
i = 2	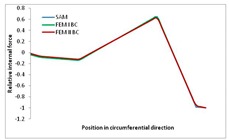	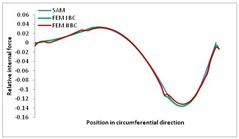	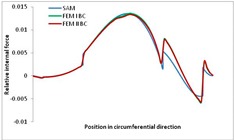
i = 3	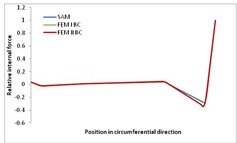	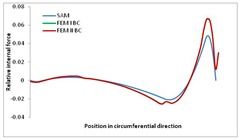	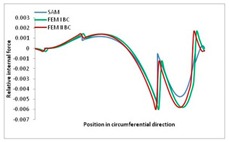
i = 4	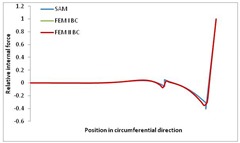	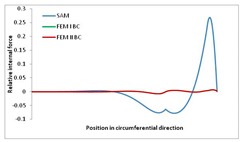	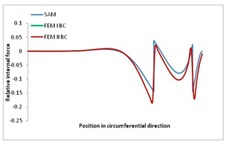

**Table 8:** The distribution of internal forces *N_x_, N_y_* and *N_xy_* for the LC–2 beam.

Index	*N_x_*	*N_xy_*	*N_y_*
i = 1	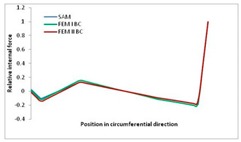	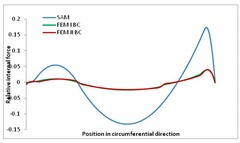	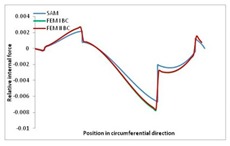
i = 2	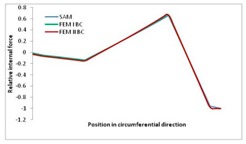	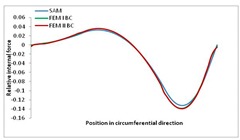	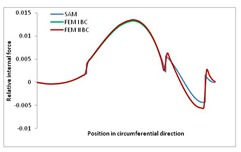
i = 3	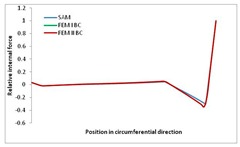	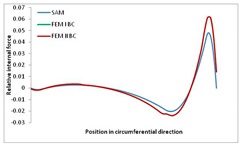	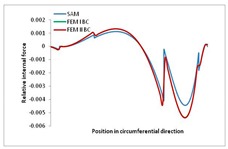
i = 4	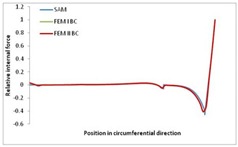	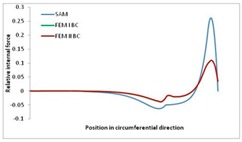	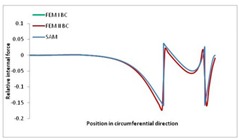

**Table 9:** The distribution of internal forces *N_x_, N_y_* and *N_xy_* for the LC–3 beam.

Index	*N_x_*	*N_xy_*	*N_y_*
i = 1	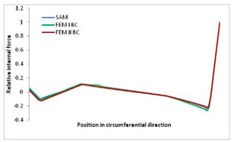	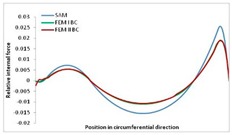	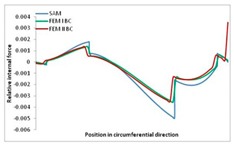
i = 2	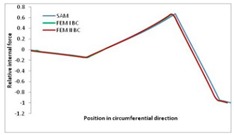	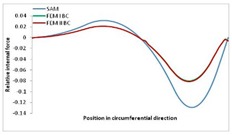	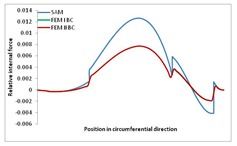
i = 3	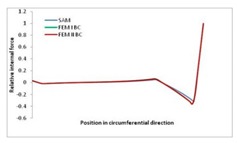	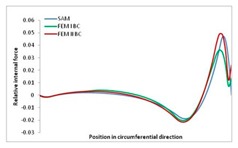	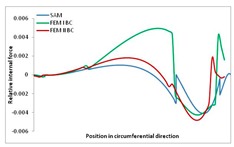
i = 4	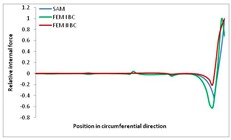	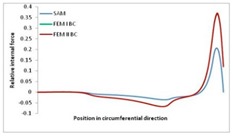	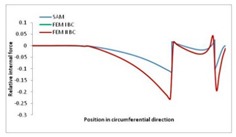

**Table 10:** Primary and secondary coefficients *a_pqr_* for LC–1, LC–2 and LC–3.

3 Mode Approach	Coefficients	*a_pqr_*		
		LC–1	LC–2	LC–3
1, 2, 3	primary	*a_211_* (ζ_2_ ζ _1_^2^)	*a_211_* (ζ_2_ ζ _1_^2^)	*a_211_* (ζ_2_ ζ _1_^2^)
	secondary	*a_133_* (ζ_1_ ζ _3_^2^)	*a_133_* (ζ_1_ ζ _3_^2^)	–
1, 2, 4	primary	*a_144_* (ζ_1_ζ _4_^2^) *a_244_* (ζ_2_ ζ _4_^2^)	*a_144_* (ζ_1_ζ _4_^2^) *a_244_* (ζ_2_ ζ _4_^2^)	*a_144_* (ζ_1_ζ _4_^2^) *a_244_* (ζ_2_ ζ _4_^2^)
	secondary	*a_211_* (ζ_2_ ζ _1_^2^)	*a_211_* (ζ_2_ ζ _1_^2^)	*a_211_* (ζ_2_ ζ _1_^2^)

**Table 11:** Dimensionless coefficients I1, I2 and I3 for LC–1, LC–2 and LC–3.

3 Mode Approach	LC–1			LC–2			LC–3		
	I1	I2	I3	I1	I2	I3	I1	I2	I3
1, 2, 3	0.05	1.001	0.95	0.02	1.04	0.97	0.02	1.0001	0.97
1, 2, 4	0.06	1.005	0.93	0.04	1.004	0.95	0.04	1.004	0.95

**Table 12:** Load-carrying capacity for FRP beam.

Methods	LC–1		LC–2		LC–3	
	*M_s_/M*_min_ (1,2,3)	*M_s_/M*_min_ (1,2,4)	*M_s_/M*_min_ (1,2,3)	*M_s_/M*_min_ (1,2,4)	*M_s_/M*_min_ (1,2,3)	*M_s_/M*_min_ (1,2,4)
FEM BC I	0.974	0.989	0.958	0.927	0.886	0.885
FEM BC II	0.993	0.986	0.913	0.922	0.888	0.874
SAM	0.843	0.923	0.796	0.823	0.827	0.839

**Table 13:** Equilibrium paths for interactive buckling of FRP beam.

LC–1	LC–2	LC–3
Interaction of mode i = 1, 2, 3	Interaction of mode i = 1, 2, 3	Interaction of i = 1, 2, 4
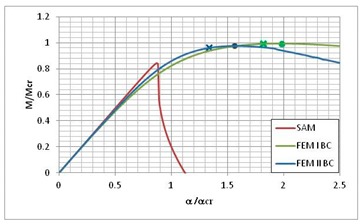	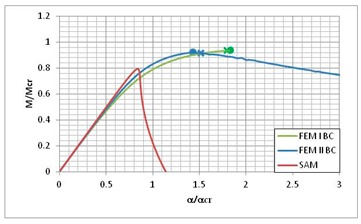	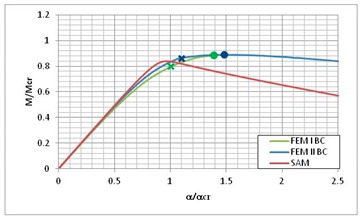

**Table 14:** Maximum bending moments corresponding to the destruction of the composite layer for the FRP beam.

Methods	LC–1		LC–2		LC–3	
	*M_H_/M*_min_ (1,2,3)	*M_H_/M*_min_ (1,2,4)	*M_H_/M*_min_ (1,2,3)	*M_H_/M*_min_ (1,2,4)	*M_H_/M*_min_ (1,2,3)	*M_H_/M*_min_ (1,2,4)
FEM BC I	0.972	0.980	0.958	0.913	0.873	0.798
FEM BC II	0.960	0.972	0.913	0.914	0.843	0.863

**Table 15:** Internal forces in LC–1 beam under the load corresponding to the first failure and the load-carrying capacity.

LC–1	*N_x_*	*N_xy_*	*N_y_*
Interaction i = 1,2,3	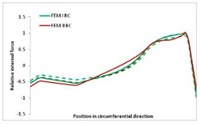	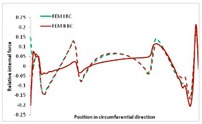	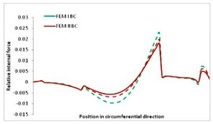
Interaction i = 1,2,4	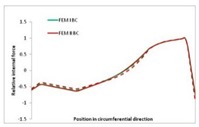	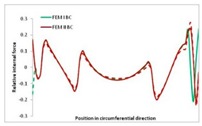	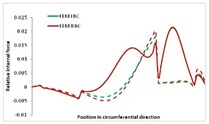

**Table 16:** Internal forces in LC–2 beam under the load corresponding to the first failure and the load-carrying capacity.

LC–2	*N_x_*	*N_xy_*	*N_y_*
Interaction i = 1,2,3	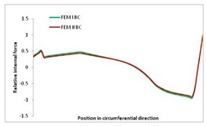	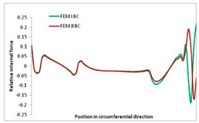	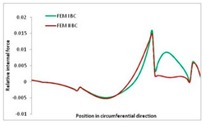
Interaction i = 1,2,4	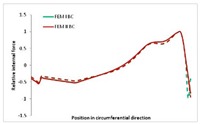	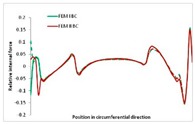	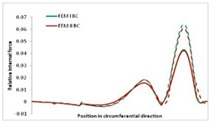

**Table 17:** Internal forces in LC–3 beam under the load corresponding to the first failure and the load-carrying capacity.

LC–3	*N_x_*	*N_xy_*	*N_y_*
Interaction i = 1,2,3	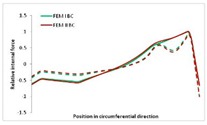	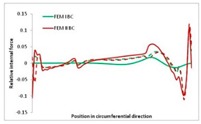	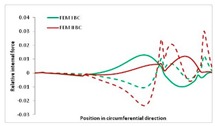
Interaction i = 1,2,4	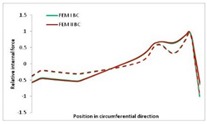	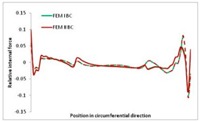	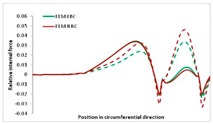

## Appendix A

The non-linear three index coefficients of the first approximate system (5) of non-linear equations are determined in accordance with (6a), as follows:

(A1) $$a_{r}={\bar{a}}_{pqr}/{\bar{a}}_{r}$$

where:

(A2) $${\bar{a}}_{r}=-\lambda_{r}\sigma_{0}\cdot l_{2}(U_{r})$$

(A3) $${\bar{a}}_{pqr}=\sigma_{p}\cdot l_{11}(U_{q},U_{r})+0.5\sigma_{r}\cdot l_{11}(U_{p},U_{q})$$

These coefficients are determined based only on linear eigenvalues. This is an important observation simplifying solutions to problems limited to the first approximation. Coefficients *ā_pqr_* (A3) are determined from the following integral relationships:

(A4) $$\begin{array}{l} \sigma_{p}\cdot l_{11}(U_{q},U_{r})=\sum_{i=1}^{n}\iint [N_{px}(u_{q,x}u_{r,x}+v_{q,x}v_{r,x}+w_{q,x}w_{r,x})+N_{py}(u_{q,y}u_{r,y}+v_{q,y}v_{r,y}+w_{q,y}w_{r,y})\\ +N_{pxy}(u_{q,x}u_{r,y}+u_{q,y}u_{r,x}+v_{q,x}v_{r,y}+v_{q,y}v_{r,x}+w_{q,x}w_{r,y}+w_{q,y}w_{r,x})]dxdy=IX+IY+IXY \end{array}$$

where the abbreviated designation of integer expressions corresponding to the internal forces is introduced:

(A5) $$IX=\sum_{i=1}^{n}\iint \left[N_{px}(u_{q,x}u_{r,x}+v_{q,x}v_{r,x}+w_{q,x}w_{r,x})\right]dxdy$$

(A6) $$IY=\sum_{i=1}^{n}\iint \left[N_{py}(u_{q,y}u_{r,y}+v_{q,y}v_{r,y}+w_{q,y}w_{r,y})\right]dxdy$$

(A7) $$IXY=\sum_{i=1}^{n}\iint \left[N_{pxy}(u_{q,x}u_{r,y}+u_{q,y}u_{r,x}+v_{q,x}v_{r,y}+v_{q,y}v_{r,x}+w_{q,x}w_{r,y}+w_{q,y}w_{r,x})\right]dxdy$$

and *n*—the number of component plates.

As is evident, the internal forces correspond only to the buckling modes marked with the index *p*, while the displacement derivatives correspond to the buckling modes marked with indices *q, r*.
